# Strengthening of 3D Printed Fused Deposition Manufactured Parts Using the Fill Compositing Technique

**DOI:** 10.1371/journal.pone.0122915

**Published:** 2015-04-16

**Authors:** Joseph T. Belter, Aaron M. Dollar

**Affiliations:** Department of Mechanical Engineering and Material Science, Yale University, New Haven, Connecticut, United States of America; Università di Trento, ITALY

## Abstract

In this paper, we present a technique for increasing the strength of thermoplastic fused deposition manufactured printed parts while retaining the benefits of the process such as ease, speed of implementation, and complex part geometries. By carefully placing voids in the printed parts and filling them with high-strength resins, we can improve the overall part strength and stiffness by up to 45% and 25%, respectively. We discuss the process parameters necessary to use this strengthening technique and the theoretically possible strength improvements to bending beam members. We then show three-point bend testing data comparing solid printed ABS samples with those strengthened through the fill compositing process, as well as examples of 3D printed parts used in real-world applications.

## Introduction

While the quality of additive manufacturing (AM) technologies has improved drastically over the past few decades, one of the major limitations to the wider-spread implementation of 3D-printed components continues to be the limited strength of printed parts. This limitation is in large part due to the small number of materials that are currently available and compatible with existing technologies. While processes compatible with metals such as Selective Laser Sintering (SLS) are becoming more robust and widespread, their costs are likely to remain much higher than other processes such as Fused Deposition Modeling (FDM) and Stereolithography (SLA). FDM in particular has caught hold in the hobbyist and do-it-yourself communities with the availability of low-cost machines that are approaching the part quality capabilities of commercial machines. However, the available materials are generally limited to ABS, PLA, Nylon, and Polycarbonate, with bulk strengths between 30–100 MPa and elastic moduli in the 1.3–3.6 GPa range (Table 1) [1–5], with those numbers greatly reduced in printed components [5].

**Table 1:** Bulk Material Properties of common FDM printed materials and Casting Resins.

*Material*	*Tensile Strength(MPa)*	*Flexural Strength(MPa)*	*Flexural Modulus (MPa)*
ABS-P430 [2]	37.0	353.0	2,250
PLA-3052D [3]	62.0	3108.0	3,600
Nylon 12 [4]	48.3	369.0	1,310
Polycarbonate [5]	68.0	3104.0	2,234
Urethane 305 [14]	20.7	327.6	813
Urethane IE-3076 [15]	72.4	3117.2	2,896
Epoxy 105/205 [16]	54.5	397.2	3,178

**Fig 1:**
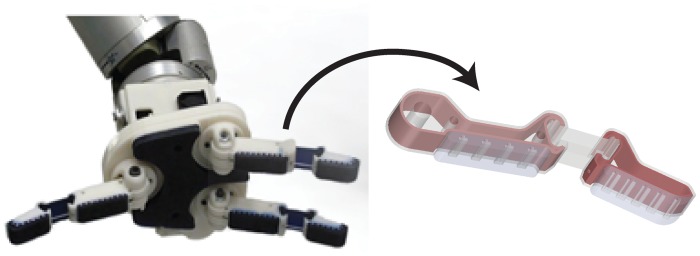
Fingers of the i-HY hand are made using fill compositing to add strength to the 3D printed components. The red (dark) portion illustrates the internal reinforcing structure of the 3D printed part.

In this paper, we discuss one method for greatly improving the mechanical strength of 3D printed components, via compositing with higher-strength resins filled into voids printed within the structure. The approach retains 3D printing’s benefits of fast and easy construction and the ability to make complex geometries, while only requiring a few straight-forward and easy to implement post-processing steps. Using FDM as a platform, we examine a number of different options for printing parts that can be filled with resins after printing, including hollow parts, sparse-filled prints, and prints with hollow channels oriented to maximize strength-to-weight ratio, and experimentally evaluate the changes in strength and stiffness via an ASTM standard three-point bend test (ASTM-D790 [6]). We further demonstrate the concept to improve strength in three practical applications: a spoked wheel, robotic finger link, and standard open-end wrench. The general process is illustrated in Fig 1.

Related to improving the strength of 3D printed components, a number of related works exist. Indirectly related to the proposed work, researchers have developed intricate software solutions to enhance the strength of 3D printed structures through the addition of ribs and internal printed supports [7], but these approaches are still limited by the strength of the material being used and FDM print orientation. Even systems that attempt to optimize the print extrusion, temperature, and between layer bond strength are still limited by the strength of the thermoplastic and offer only marginal improvements over standard FDM printing methods [8]. 3D printing can also be useful in creating molds that are later used to cast components from stronger materials [9, 10]. This way, some of the advantages of 3D printing can still be utilized and result in a stronger component and made from a wider variety of materials. However, casting of components can place limitations of part detail and overall geometry depending on the complexity of the mold.

There are numerous efforts to improve the properties of materials available for AM processes. These range from improvements in material chemistries [11], to composite material feedstocks such as metal-polymer composites [12] and carbon fiber-reinforced materials [13]. No published work has been found that investigates the concept described in this paper: printing AM parts with voids that can be filled with resins in order increase the overall part strength.

In the following section, we present an overview of the strength limitation of 3D printed materials. We then discuss how the proposed “fill compositing” technique can theoretically improve bending stiffness and strength. We present the results of flexure testing to show the investigation of fill-composite parameters such as infill type and resin material and their roll in increasing the overall part strength. Finally, we show that this technique can be used to strengthen common components including a spoked wheel, robotic finger link, and standard open-end wrench.

## Strength of 3D Printed Materials

### A. Common FDM printed materials

FDM based 3D printing relies on fusing sequential layers of material extruded from a small nozzle to form the overall part geometry. Due to this process, the available materials are currently limited to thermoplastics although additional materials with additives and blends are being investigated [17]. Table 1 shows the strength of the raw bulk materials most commonly used in FDM. These materials are used in the popular Stratasys and Makerbot brand FDM printers. As a comparison, three additional materials are shown in Table 1 including two common casting urethanes [14,15] and a common two-part Epoxy resin [16]. It is important to note that these are bulk properties and do not represent the properties of the material when 3D printed through FDM.

### B. Strength of materials as printed

The FDM printing method deposits fibers/beads of thermoplastic in two-dimensional layers, building up the layers on top of each other to form the desired part geometry. The layering and direction of the fibers introduces an anisotropic effect that greatly influences the overall strength of the 3D printed part [18, 19, 20]. Numerous researcher have shown that FDM printed materials show an approximate 45% decreases in modulus when compared to the bulk material [5]. Smith et al. also showed a 30–60% decrease in ultimate tensile strength based on part orientation when comparing the FDM printed test samples with the bulk material properties [5]. Careful tuning of the printer parameters including extrusion rates, bead sizes, and temperatures can also be performed to improve part strength although these techniques are still bounded by anisotropic behaviors and the bulk properties of the printed thermoplastic [8].

**Fig 2:**
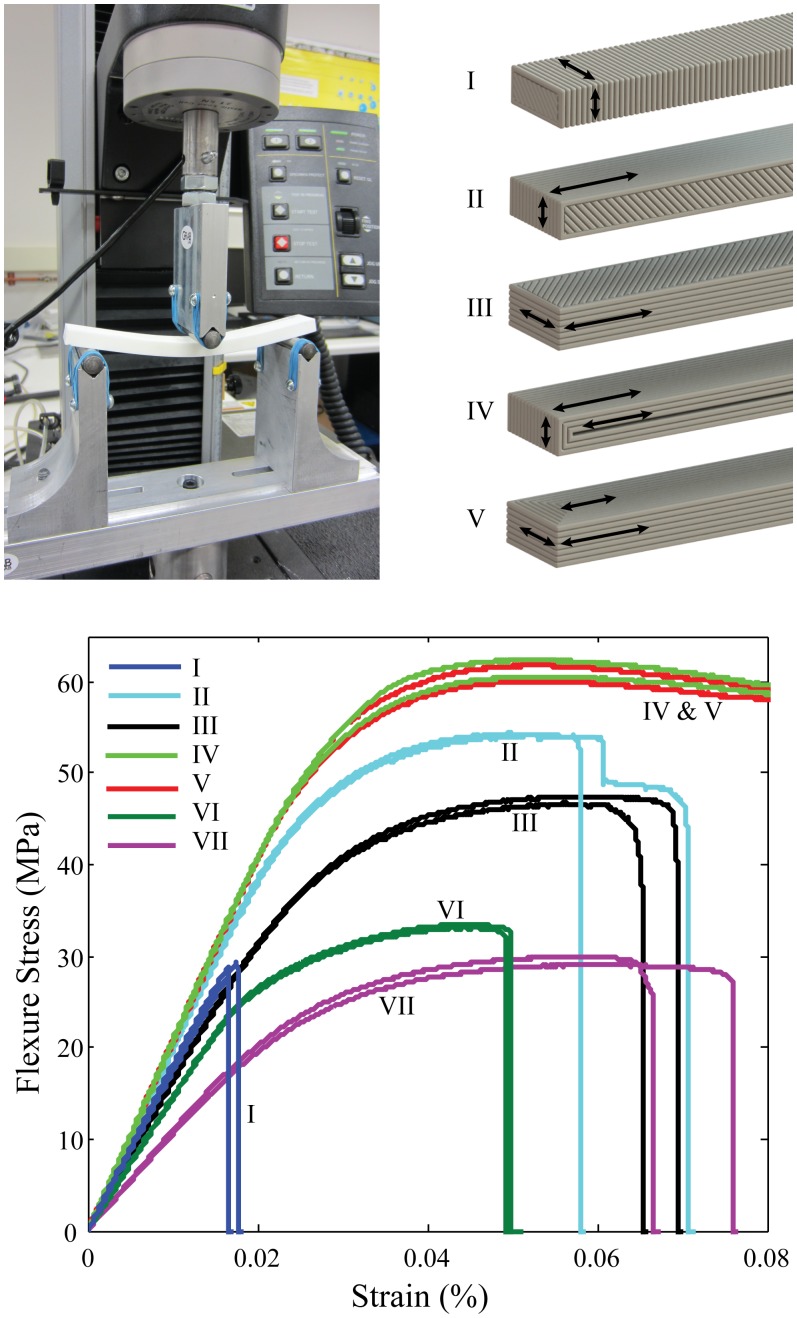
ABS material exhibits a large variation in flexure strength based on print orientation and printer parameters. I) upright print with raster infill, II) vertical print with raster infill, III) horizontal print with raster infill, IV) vertical print with multiple contours, V) horizontal print with multiple contours, VI) sparse-fill vertical print, VII) sparse-fill horizontal print.

To verify the effects of FDM print orientation on overall part strength, we conducted three-point bend flexure testing of printed samples. The testing procedure and sample preparation, as shown in Fig 2 (top left), is detailed in the section titled “Flexural Testing of ‘Fill-Composite’ Samples”. All tested samples were printed from ABS-P430 [2], on a Fortus-250m printer. Using the same generic rectangular sample geometry, we used the Insight software (provided by Stratasys) to print in various build orientations relative to the printer build tray. By default, the software builds the part using a single outer contour pass and then an internal raster to fill each sequential layer completely with ABS material. The raster angle of each layer is altered by 90 degrees in an attempt to give a more uniform solid structure. Other options can be selected that allow the internal sections of the part to be printed in a sparse/less dense packing of extrusion paths. The outer contour can also be altered so that the part is printed with multiple contours from the outside of each layer inward which eliminates the need for the raster fill of each layer. The build orientation and extrusion path parameters all affect the orientation of the ABS fibers within the part and therefore have an influence on overall part strength. Although the samples were printed in various orientations, all samples underwent flexure testing in the orientation as demonstrated in Fig 2. A diagram of the printed fiber orientations is also shown to illustrate the difference in the printed samples.

It can be seen from the flexure stress curves in Fig 2, fibers oriented in the direction of stress (lengthwise down the sample) leads to greater overall strength. The samples with the layers oriented perpendicular to the direction of the stress, as in sample I from Fig 2, showed over 50% reduction in flexure stress as compared to sample IV and sample V which have all the fibers oriented parallel to the direction of stress in the rectangular bar sample. Although for some components, the unfavorable print orientations (such as the vertical print direction depicted as sample I) cannot be avoided if it is required that the part be stressed along multiple orientations. In this case, the component strength is limited by the weakest print orientation and should be considered when designing the component with the intent of manufacturing through FDM methods.

## Fill Compositing Technique

**Fig 3:**
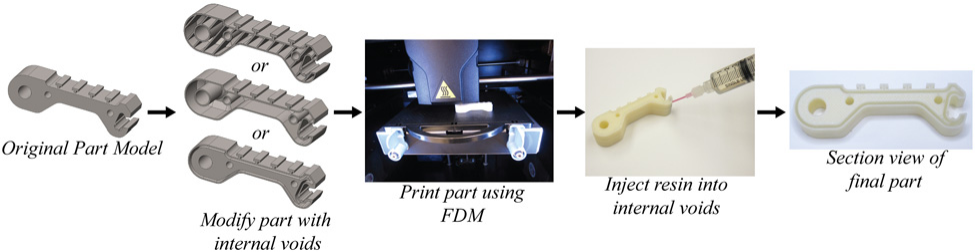
The process of fill compositing uses the original part geometry but takes advantage of voids designed into the printed component which are filled with higher-strength resin. The process is illustrated here with the proximal link of the i-HY [28] robot finger.

By utilizing hollow voids and channels printed internally to the components as molds for casting materials, complex internal reinforcing structures can be made that provide an increase in part strength and stiffness. Although the bulk material properties of common casting materials including urethane and epoxy do not far exceed those of the bulk 3D printed material, as shown in Table 1, their properties are isotropic when molded and therefore do not exhibit the same orientation preferences as 3D printed materials. The process of strengthening a 3D printed part with the fill compositing technique is illustrated in Fig 3. Each of the three methods will be discussed in the following sections.

### A. Placing hollow voids within the part

In Fig 3 (far left) we show the original design of the proximal link of a robot finger. There are three methods for introducing hollow voids within the printed part. The first and simplest method is to print the part using a sparse infill technique. As long as the sparse infill is porous enough to allow resin to fill the cavity, the resin will take up the hollow volume in the part. The simplicity of this method is that all modification can be done in the 3D printer slicing software and no changes to the original part geometry are required.

The second way to modify the part is to make the internal portion of the component completely hollow. The external walls of the part act as a mold to internally cast the stronger resin material. This technique can be thought of as using FDM 3D printing to create a mold where the mold remains to provide the detailed outer geometry. The limitations to the hollow structures are based on the ability for the printer to create these voids without the need for support material. Factors related to the specific printer including overhang angle, unsupported span length, and minimum wall thickness all relate to the necessity for support structures. Using both a Stratasys uPrint and Stratasys Fortus-250m, the authors have successfully printed overhangs at a 30 degree angle from horizontal and unsupported horizontal spans of up to 3mm without the need for support material. A 0.6 mm wall thickness has shown to be sufficient to create non-porous interior and exterior layers when using FDM.

Perhaps a more appropriate and efficient method is to insert connected hollow regions that will best provide structural enhancement to the part based on the expected loading. Since the part will be 3D printed using FDM, these channels can be quite elaborate and complex. For example, if a 3D printed part needs additional strength between specific attachment features or bolt holes, hollow voids can be designed to specifically strengthen these areas without requiring the entire part to be hollow. Parts designed with this method have the highest strength to weight ratio since the injected resin is utilized in the appropriate locations.

The FDM process makes it possible to create completely void internal structures. Other types of printing, including Z-Corp powder binding and PolyJet UV curing, cannot print overhangs without support material and therefore cannot produce completely hollow voids internal to the part. It may be possible to remove the powder or soluble support from the internal voids in the part but proves to be extremely difficult for more complex geometries.

### B. Casting resin material into voids

The modified parts are printed with internal hollow sections and the detailed external geometry provided by the 3D printer. A 1mm hole is drilled into the component to access the hollow cavity(s). As shown in Fig 3, a syringe is used to inject resin into the void. The injection site should be chosen to allow for the epoxy or other casting material to set without leaking out the infill hole. Since air may become trapped in the internal voids, it is sometimes preferred to create multiple fill ports or tiny vent holes.

### C. Final part features

In the finished part, hardened resin provides structural reinforcement to the component from the inside. All external geometries of the original part are unchanged. The process can be compared to investment casting where the component provides the mold for the internal reinforcing cast structure or even overmolding where a thin plastic layer covers a strong internal structure.

## Expected Strength Improvement of Fill-Composite Parts

**Fig 4:**
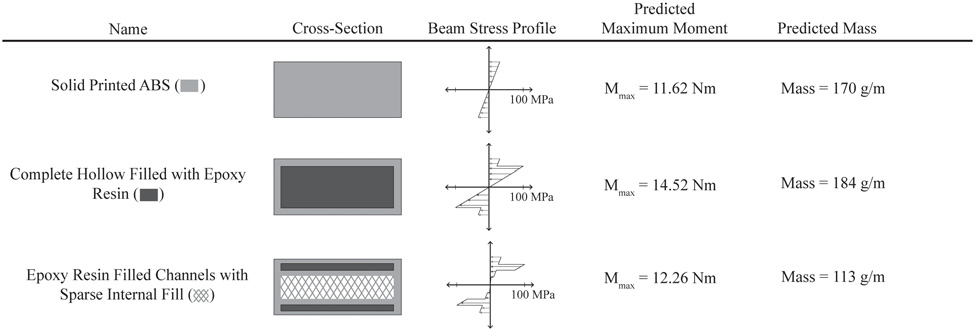
The calculated maximum bending moment for the various fill-composite cross-sections shows the ability to increase the capable bending load by 25% or reduce the mass of the beam by 33% using fill compositing with Epoxy resin.

The proposed technique creates a composite component that can leverage the added strength of the injected resin. The cross-section of the constructed samples can be analyzed to determine the effect of the added resin on the overall bending strength. Using the flexure strength properties of ABS (53.0 MPa) and Epoxy Resin (97.2 MPa) shown in Table 1, we can calculate the bending moment at failure using standard beam bending equations for each of the types of fill compositing described in the previous section. Fig 4 shows the cross-sections and associated beam stress profile for hollow filled samples and resin filled channels as compared to a standard solid printed ABS beam when subjected to three-point bending. The geometry is identical to the tested samples described in the following section. The results indicate that, for this geometry, we can expect a 25% improvement in capable bending loads through using the complete hollow filled with epoxy resin and a 5% improvement in strength with the epoxy filled resin channel geometry. However, the channel geometry shows that the bending strength can be maintained while reducing the overall beam mass by 33%. Here, *M*_max_ is the maximum bending moment before failure.

## Flexural Testing of ‘Fill-Composite’ Samples

Three-point bend testing was performed to verify and quantify the increase in strength of components produced with the fill compositing technique described earlier. Testing was also performed to determine the strongest method of fill compositing parts that contain various infill patterns/techniques. The testing was performed according to ASTM-D790 [6] using an Instron material testing system. An illustration of the testing setup is shown in Fig 2 (top left). The loading was applied at 0.1 mm/mm/min to avoid load-rate effects.

The flexural bend test samples were simple blocks of 8.3x 19.1 x152.4mm and were sized in accordance to the ASTM-D790 standard. All parts were printed with the same ABS-P430 material on the same Fortus-250m printer using Insight 9.0 software. Uncolored material was used to eliminate the effects of colorant within the ABS. With the same external part geometry, samples were prepared using the fill compositing technique including various sparse infill techniques; completely hollow printed shells filled with resin, and carefully designed resin filled channels. Control samples were also tested including cast samples of all the resin materials used, and samples of identical geometry to the resin filled channels with printed ABS in place of the epoxy. Where possible, the print orientation of the samples was varied to show the anisotropic behavior of the samples even with the resin fill.

The flexural stress, strain, and flexural modulus was calculated according to Eqs 1, 2 and 3, which assumes small angle deformation of the three-point bend specimen [21].

$$\sigma =\frac{3FL}{2bd^{2}}$$ (1)

$$\varepsilon =\frac{6dv}{L^{2}}$$ (2)

$$E=\frac{FL^{3}}{4vbd^{3}}$$ (3)

Here, *σ* is the flexure stress, *ε* is the strain, *E* is the flexure modulus, *F* is the force on the center of the beam, *L* is the span of the test setup (124.0 mm), *b* is the width of the sample (19.1mm), *d* is the sample thickness (8.3mm),and *v* is the deflection at the center of the beam. The parameters can also be normalized by the density of each sample to give a strength-to-weight ratio or stiffness-to-weight ratio.

## Results of ‘Fill-Composite’ Flexure Testing

### A. Resin Material

Although there are numerous resins that could be used to inject into the hollow voids in the printed parts, the authors wanted to test standard two-part resins that are readily available at a relatively low-cost. For this reason, we choose two common urethane resins and a standard two-part epoxy resin. The bulk strength properties of these materials exceed those of bulk ABS as shown in Table 1. The first urethane is a Smooth-cast 305 resin used by hobbyist and model makers [14]. The second urethane, is a stronger and harder, IE-3076 [15]. The final resin tested was West Marine 105–206 resin and slow-hardener typically used in the construction of fiberglass laminates [16].

**Fig 5:**
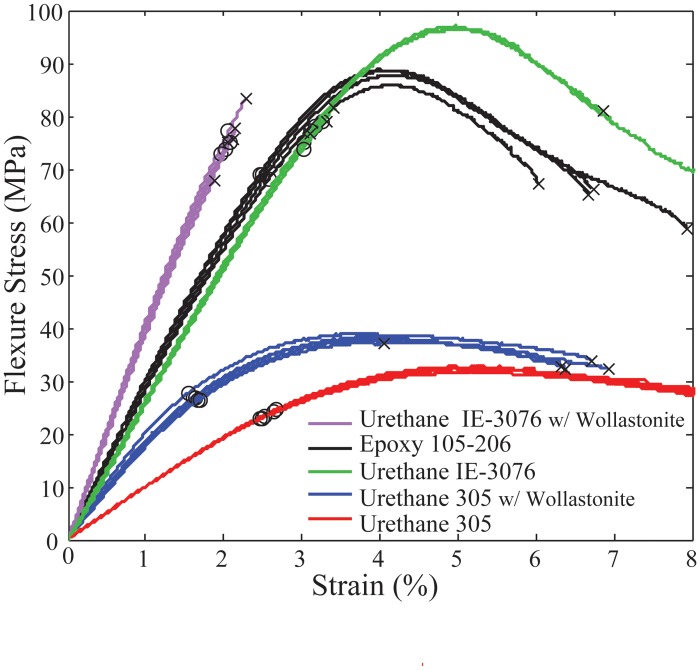
Flexure stress comparison of three common resins with and without wollastonite additive. The black x indicates the location of failure and the black circle represents the 0.2% yield strength.

In addition to the stronger resins, additives were also investigated that improve resin strength and stiffness. Both short and long chopped glass fibers were tested in both the epoxy and the urethanes but did not significantly change the flexure strength of the tested samples. A 20% by weight addition of wollastonite (as suggested by the material manufacturer) was shown to greatly improve the stiffness of the urethane samples [22]. Fig 5 shows the flexure stress of the bulk materials cast into samples of the same geometry as described in the section “Flexural Testing of ‘Fill-Composite’ Samples”. The addition of wollastonite improved the flexure stiffness of both urethanes resins tested. Although adding chopped glass fibers to the bulk epoxy greatly increased the viscosity of the resin, a 20% by weight addition of wollastonite to the urethane materials proved to maintain a low enough viscosity to inject into the voids within the printed parts. As recommended by the material manufacturer, the resins were degassed in a vacuum chamber prior to injecting them into the 3D printed parts. During curing, the parts were placed into a pressure chamber at 60 psi to minimize bubble formation within the resin. We can see from Fig 5 that of the materials tested, the Urethane IE-3076 with 20% wollastonite proved to be the stiffest with unfilled IE-3076 providing the highest overall ultimate tensile strength.

### B. Sparse Infill Parameters

The typical motivation for using sparse fill in 3D printing is to reduce the print duration or to reduce the amount of material used in a part. However, sparse fill can be used to make structures porous allowing parts to be strengthened by injection of resins through fill compositing. This method will allow any solid-filled part to be strengthened by injection without the need to redesign the part or change any of its geometries. The usual default sparse fill settings in standard 3D printing software have two distinct features. The first is that the path lines for the fill are further spaced apart than a normal dense fill and these lines are kept parallel to each other. The second feature is that every layer alternates the angle of the fill lines by 90 degrees, creating a cross hatch pattern throughout the inside of the part. While these settings are appropriate for reducing the print time and saving material, they may not be the most desirable to be used in conjunction with the fill compositing technique.

An alternate sparse fill setting “hexagonal porous” (as labeled by the Stratasys Insight software) will provide a porous inner sparse fill by stacking hexagons and other polygons layer by layer. The software will generate two alternating sets of paths to ensure that the infill is porous. Although this infill does allow for easy filling when using the fill compositing technique, the sheets and layers of ABS create shear planes within the part. Since the effects of the ABS failure planes was seen in both the default and hexagonal sparse infill samples, the authors designed a custom infill technique that would prevent the introductions of shear planes within the part and maintain adequate support to print in any geometry.

**Fig 6:**
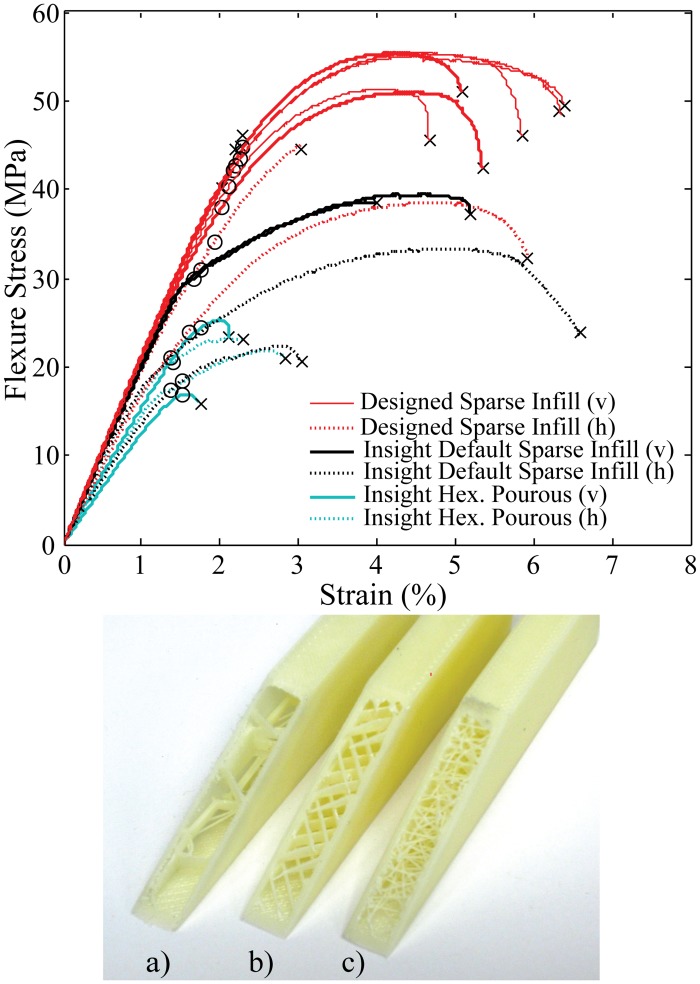
Flexure strength of 105–206 epoxy filled samples printed with various types of sparse infill. The black x indicates the location of failure and the black circle represents location of 0.2% yield strength. a) Insight hexagonal porous infill, b) Insight default sparse infill c) Designed sparse infill. The (v) or (h) indicates if the part was printed in the vertical or horizontal orientation.

To illustrate the importance of the sparse fill method, flexure testing was performed on samples with the three sparse fill techniques mentioned. To focus on the effects of the sparse infill method used, all samples were filled with West Systems 105–206 epoxy resin. Each set of samples contains parts that were printed in both the horizontal and vertical orientation. As is evident from Fig 6, the “designed” infill method led to a 43% improvement in flexural yield strength (as defined by a 0.2% deviation from pure elastic behavior) over the default sparse infill and a 87% increase in flexural yield strength over the “hexagonal porous” infill option. It is important to note that these infill techniques have different overall ABS and Resin densities based on the amount of ABS printed within the void. Without controlling for sparse infill density, we have shown that sparse infill can still be utilized with the fill compositing strengthening method if the proper infill parameters are used.

### C. Intelligently Placed Internal Voids

When the loading conditions or locations of peak stress are known within a component, it may be beneficial to strengthen only a particular area of a part. For example, in the three-point bend samples, to optimize bending stiffness, two wide and thin hollow voids were placed at the top and bottom of the sample. Therefore, the strengthening resin is located as far as possible from the neutral axis of bending and contributes more to increasing the stiffness of the part. Since the center of the bar experiences little stress during bending, the samples could be printed with sparse infill in these regions not filled with resin (see the cross-section in Fig 4). This technique for optimizing bending samples results in a structure similar to a composite laminate with stiff outer layers and a lightweight internal core. With more complex parts, these channels can encircle the entire perimeter or be specifically tuned to the location of peak stresses within the part. If desired, more advance topology optimization techniques can be used to further improve the overall strength or stiffness of the part [23, 24]. Many of these techniques involve iterative finite element methods to determine the optimal part geometry.

### D. Comparison of Fill Compositing Techniques and Materials

**Fig 7:**
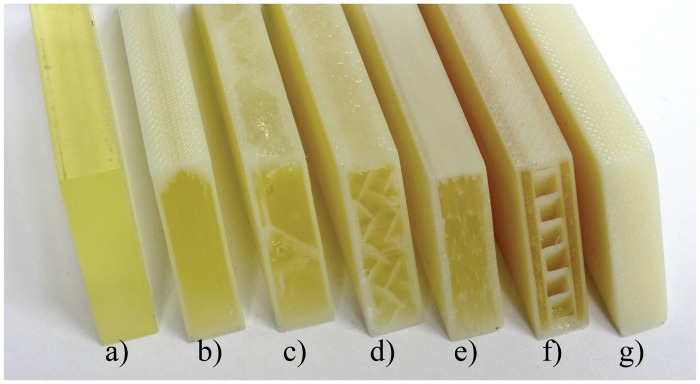
Cross-sections of the tested samples including the raw material cast samples, and the solid printed ABS samples. a) West systems 105–206 Epoxy, b) Epoxy filled hollow shell, c) Hexagonal porous infill, d) Insight default sparse infill, e) Designed sparse infill, f) Epoxy filled channels, g) solid printed ABS. All the above images are of 105–206 epoxy but the same samples were made with the IE-3076 urethane with wollastonite additive.

**Fig 8:**
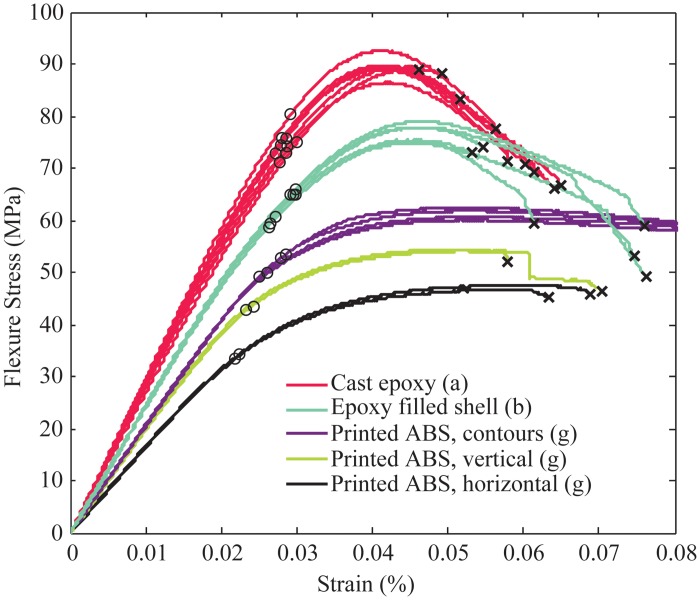
Flexure strength of epoxy filled shells made using fill compositing as compared to solid printed ABS in various orientations. The test samples are labeled according to the cross-section image in Fig 7. The black x indicates the location of failure and the black circle represents location of 0.2% yield strength.

Fig 7 shows a cross-section view of the samples that were studied to determine the best material and parameters to strengthen components using fill compositing. Fig 8 shows the flexure stress for samples of solid printed ABS at different orientations, as indicated by the letter label, compared to West Systems 105–206 epoxy resin filled samples made using fill compositing. The small black circle shows the point of 0.2% yield criteria, while the black x, indicates the location of failure for the sample. Bulk samples of epoxy are also shown as a comparison. The data shows an improvement in flexure strength and flexure modulus of epoxy filled shells as compared to all orientations of solid printed ABS. Since the print orientation is known to have a large impact on the overall component strength, we also tested samples printed in the least favorable orientation (printed upright). The results show a 60% improvement in ultimate flexure strength and an improvement in overall flexure stiffness.

**Fig 9:**
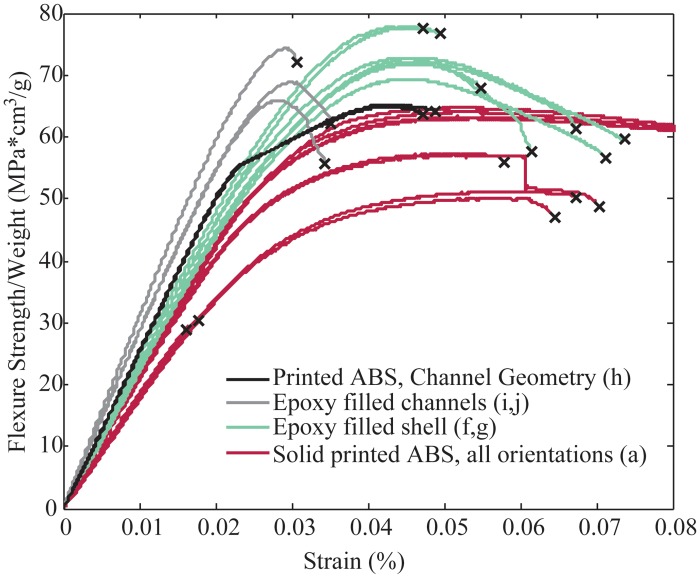
Flexure strength to weight ratio of solid ABS samples compared to those manufactured using fill compositing. As a control, samples were also tested that were printed the ABS in the same geometry as the epoxy filled channel samples.

In addition to stronger components, many robotic applications have requirements related to reduced weight or increased stiffness. The data in Fig 9 shows the results of three-point bend testing with the flexure stress normalized by the density of the sample. It can be seen that the epoxy filled shell samples have a higher overall strength to weight ratio than all print orientations of solid ABS. Also, the epoxy filled channel samples showed the highest possible stiffness to weight ratio of all samples tested with epoxy infill. This compares well to the predicted improvement in weight and strength calculated in Fig 4. As a comparison, samples were printed and tested that had the same geometry as the epoxy filled channel samples, but completely printed in ABS. This shows the effect of both the geometry change as well as the epoxy reinforcement.

The results of the three-point bend testing can be summarized by comparing the solid printed ABS samples with the epoxy filled samples over all measured material properties. Table 2, shows the strength and stiffness comparison of the samples filled with West Systems 105–206 epoxy. The flexural yield strength was evaluated at 0.2% offset from linear elastic behavior.

**Table 2:** Strength and Stiffness Comparison of Epoxy Filled Samples.

*Property*	Solid ABS, best orientation	Epoxy Filled ABS shell	Epoxy filled Designed Sparse Infill	ABS printed channel Structure	Epoxy filled channels
Flexural Yeild Strength (MPa)	50	62	43	32.3	38
Flexural Modulus (MPa)	2071	2600	2490	1475	1730
Flexural Strength/weight (Mpa*cm^3/g)	52.7	59.5	57.1	55.9	63.5
Flexural Modulus/weight((Mpa*cm^3/g)	2150	2280	2180	2540	2950

**Fig 10:**
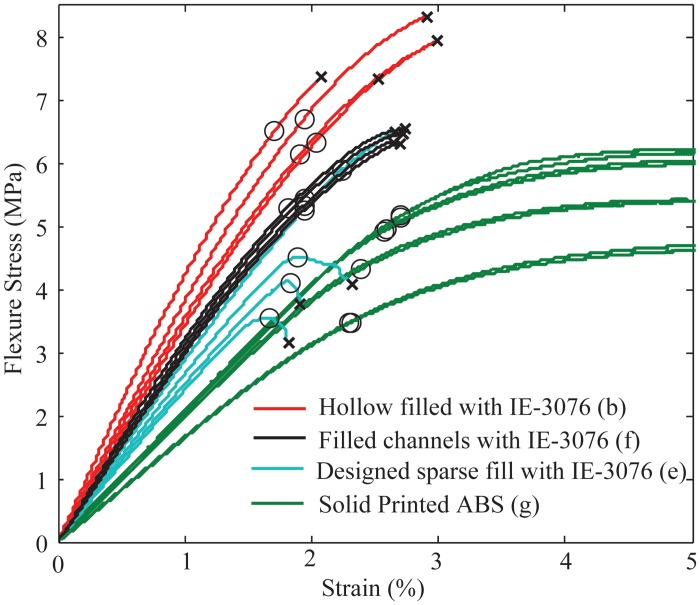
The flexure strength of the fill-composite sample using IE-3076 Urethane with wollastonite additive showed a large increase in stiffness over solid printed ABS samples. The letter labeling indicates the cross-section of the sample as illustrated in Fig 7. The solid printed ABS samples are shown for all printed orientations.

In addition to the epoxy filled samples, IE-3076 urethane with 20% by weight wollastonite additive was tested. Fig 10 shows the flexural strength comparison between the solid printed ABS samples (all print orientations), and the various fill compositing techniques strengthened with IE-3076 urethane. The IE-3076 proved to greatly increase the stiffness of the samples resulting in a 25% improvement in stiffness over the best orientation of solid ABS. The IE-3076 with wollastonite additive also increased the flexure yield strength of the samples by 30%. These results are summarized in Table 3.

**Table 3:** Strength and Stiffness Comparison of IE-3076 w/ filler Samples.

*Property*	Solid ABS, best orientation	IE-3076 Filled ABS shell	IE-3076 filled Designed Sparse Infill	ABS printed Laminate Structure	IE-3076 filled channels
Flexural Yeild Strength (MPa)	50	65	42	32.3	54
Flexural Modulus (MPa)	2071	2850	2520	1475	2410
Flexural Strength/weight (Mpa*cm^3/g)	52.7	63.2	46.2	55.9	58.1
Flexural Modulus/weight((Mpa*cm^3/g)	2150	2920	2610	2540	3180

*IE-3076 used with 20% by weight of wollastonite additive

## Strength Testing of Practical Printed Components

In the field of robotics, functional load bearing components such as robot legs, fingers, wheels, and structural frames are increasingly being fabricated using standard fused deposition manufacturing (FDM) methods [25,26], albeit with varying levels of success. Examples of robotic systems that rely nearly completely on 3D printed ABS components include the Veter robotic vehicle [27], Shady Bot [28], the Aracna quadruped platform [29], and the Yale OpenHand Project [30].

In order to demonstrate our approach in a more practical application, three practical examples were manufactured to evaluate the benefits of using fill compositing as a simple and easy method to strengthen 3D printed parts: the proximal link of a robotic finger; wheel whose spokes and outer perimeter were reinforced with the proposed method; and a standard open-end wrench.

### A. Testing Samples and Methods

**Fig 11:**
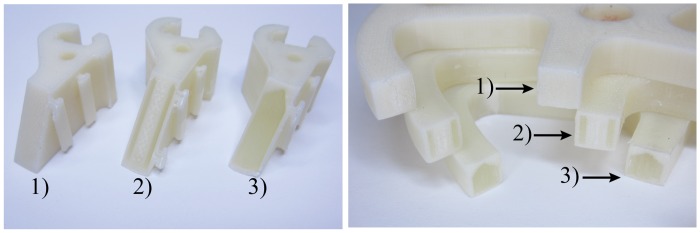
Cross-section view of the robotic components (left) proximal joint of the robot finger, (right) spokes and outer ring of the wheel. 1) Solid printed ABS, 2) 1mm channels filled with epoxy, 3) Hollow printed shell filled with epoxy.

Numerous versions of each component were created as a comparison of actual component strength. The first was a solid printed ABS sample printed in the vertical (favorable) direction with solid raster fill. The second sample was created using fill compositing with a 1mm wide channel filled with West Systems 105–206 epoxy placed just inside the entire outer perimeter of the part. For the robotic finger link, the channel was placed far enough away from the surface to maintain features used for grip pad adhesion and connection of a flexure at one end. For the wheel, the channels were placed in both the outer perimeter and spokes. The final test was to fill the entire internal cavity of all three parts with epoxy. Fig 11 shows a cross-section view of the three sample types for the proximal finger link and the wheel. You will notice in the hollow printed shell filled with epoxy, the upper surface was tapered to provide a 30 degree overhang angle since the span was too wide to bridge with the FDM printer.

**Fig 12:**
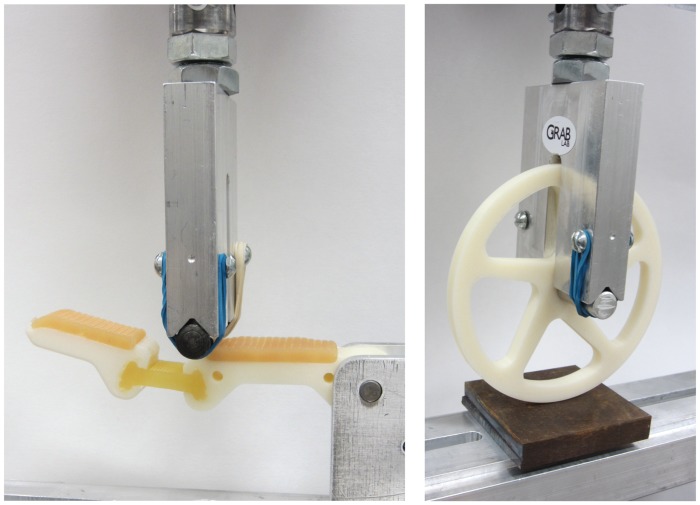
Images of testing setup on an Instron Testing system to measure failure loads of the robot finger proximal joint (left) and a simple robot wheel (right).

The overall strength of the samples was tested using a modified three-point bend fixture. For the proximal finger link, a load was placed on the distal end of the proximal linkage and applied until failure. The wheel strength was tested by applying a load to the center axle against a flat plate. All three sample types were oriented in the same spoke angle during the test as illustrated in Fig 12 (right).

### B. Testing Results

**Fig 13:**
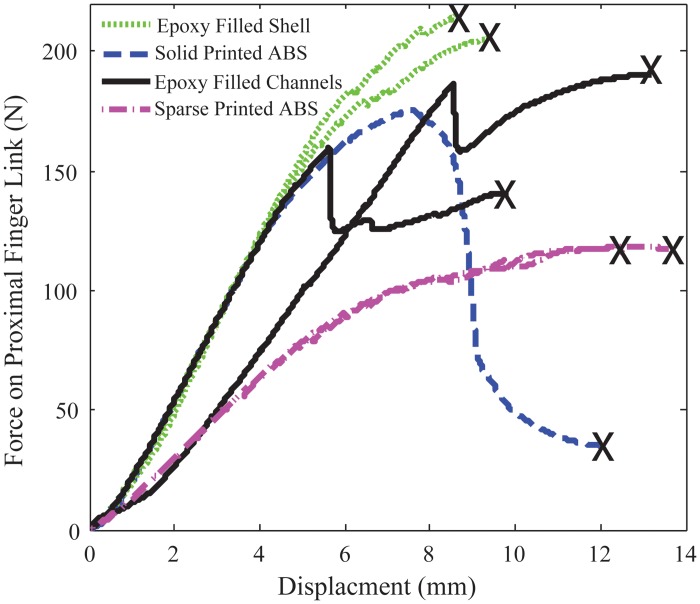
Comparison of robotic finger link strength shows improvement in failure strength using a 3D printed shell of the same part geometry filled with epoxy resin. The black x shows the point of failure.

**Fig 14:**
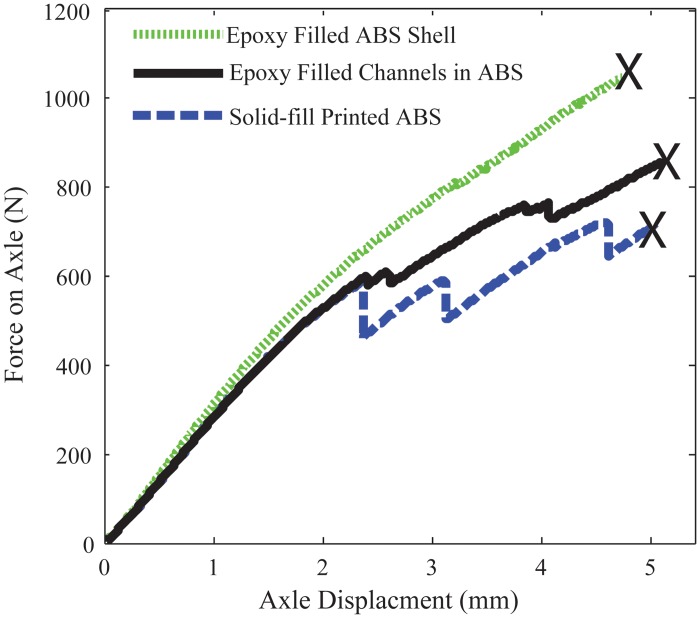
Comparison of wheel strength shows a 45% increase in load capacity using fill compositing with epoxy resin versus a solid printed ABS component. The black x shows the point of failure.

Due to the complex geometry being tested, we will directly compare the failure load instead of failure stress as was done in the standard three-point bend tests. Fig 13 shows the comparison of proximal robot link strength (bending in the extension direction) of the three samples types. Fig 14 shows the comparison of robot wheel strength between solid printed samples and those filled with resin. These plots also show the relative stiffness of the three samples by analyzing the slope of the force- displacement curve.

**Fig 15:**
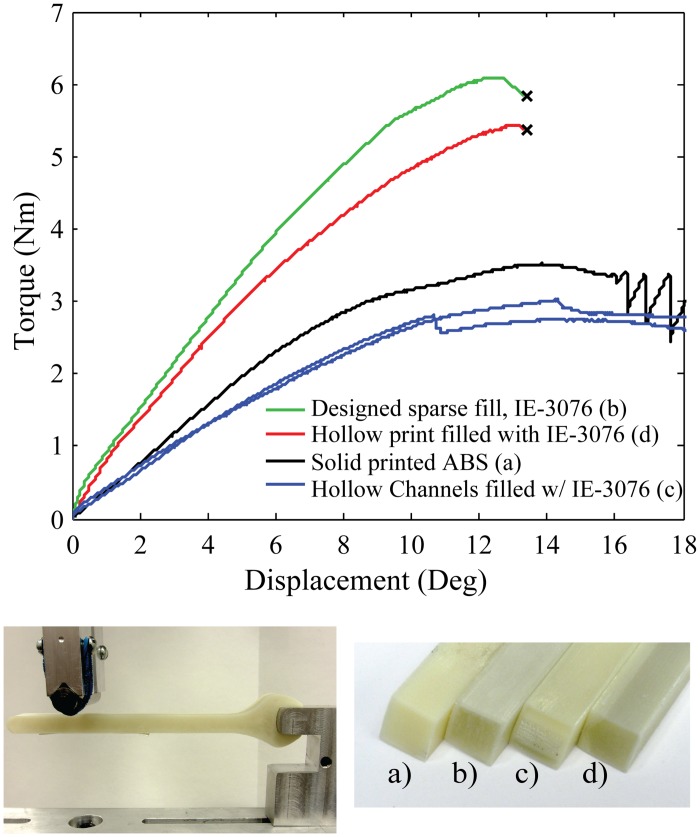
The cross-sections of a printed open-end wrench that have been strengthened with fill compositing are shown in the bottom right. a) Solid printed ABS, b) Designed sparse fill with IE-3076 with wollastonite additive, c) Hollow channels filled with IE-3076 with wollastonite additive, d) Hollow print filled with IE-3076 with wollastonite additive. The top plot shows the torque and rotational displacement of each sample during destructive testing.

The final wrench component was filled with IE-3076 with wollastonite additive as shown in Fig 15. The torque on the end of the wrench was measured as a function of the angular displacement of the wrench around the stationary simulated nut. The results of the component tests are best summarized in Table 4.

**Table 4:** Strength Comparison of Robotic Components.

*Property*	Solid ABS orientation (a)	Resin Filled ABS shell (c)	Resin filled channels (b)
**Finger Link**			
Peak Force (N)	175	208	190
Stiffness (N/mm)	30.6	32.7	26.1
**Wheel**			
Peak Force (N)	720	1048	895
Stiffness (N/mm)	268.7	303.7	269.5
**Wrench**			
Peak Torque (Nm)	33.5	52.1	28
Stiffness (Nm/deg)	0.37	0.58	0.31

## Discussion

We have shown that we can enhance the strength of 3D printed components above the capabilities of the solid printed material even in the most preferable print orientation. The process of fill compositing is simple and takes advantage of the benefits of low-cost FDM printing. This has a direct effect on the future use of 3D printed parts in the rapid development of functional load bearing components.

In the three-point bend samples, the overall yield strength of a simple printed hollow structure filled with epoxy resin was 24% higher than the most preferable solid ABS print orientation. The stiffness was also 25% higher with the epoxy filled samples. One of the greatest advantages was the improvement in strength and stiffness to weight ratio of 13.6% and 16.1% respectively, through the use of hollow channels designed into the part and filled with epoxy resin.

The test components also showed improved properties through the use of fill compositing. The finger link showed a 19% improvement in failure load, while the wheel showed a 45% increase in failure load. The wrench showed a more than double increase in capable exerted torque. The investigation into the preferred print orientation showed that the strength limitations of the worst print orientations can be overcome using fill compositing.

There still was a significant improvement in the strength of even the epoxy filled printed shells when the shells were printed in the preferred orientation. This shows that it is still beneficial to consider the orientation of the print fibers when using this technique to strengthen 3D printed parts.

One limitation to the fill compositing method is the necessity for the parts to be printed with non-porous internal voids. Some FDM printer settings will create porous parts that do not properly block flow of the resin into sparse fill areas of the part. It is necessary to adjust printer setting, specifically with regard to raster fill and contour path overlap, to prevent porous internal cavity wall surfaces.

## Supporting Information

S1 File Fill Compositing RawData. This is the raw data and information on all the samples presented in this manuscript.
(ZIP)

S2 File Belter IROS2014. This is the preliminary conference version of this work.
(PDF)
