# Supplementary material for: Strengthening of 3D Printed Fused Deposition Manufactured Parts Using the Fill Compositing Technique
Source: PLoS One. 2015 Apr 16;10(4):e0122915. doi: 10.1371/journal.pone.0122915 (PMC4400136; doi:10.1371/journal.pone.0122915)
Supplement: S2 File — This is the preliminary conference version of this work. (PDF) [file pone.0122915.s002.pdf]

2013 IEEE/RSJ International Conference on
Intelligent Robots and Systems (IROS)
September 14-18, 2014. Chicago, IL, USA,

Strengthening of 3D Printed Robotic Parts via Fill Compositing

Joseph T. Belter and Aaron M. Dollar, *Senior Member, IEEE*

***Abstract*— Three-dimensional printing technology, also known as additive manufacturing, has shown a significant increase in popularity as the cost of printers comes down and part accuracy and build quality continually improves. To date, the major limitation of the various additive manufacturing techniques is the limited range of print materials and properties, with 3d printed parts unable to be used in most load-bearing applications in robotics and other domains. In this paper, we present a technique for increasing the strength of 3d printed parts while retaining the benefits of the process such as ease and speed of implementation and complex part geometries. By carefully placing voids in the printed parts, which are later filled with higher-strength resins, we can improve the overall part strength and stiffness by up to 45% and 25%, respectively. We show three-point bend testing data comparing solid printed ABS samples with those strengthened through the fill compositing process, as well as examples of 3D printed parts used in robotic applications.**

I. Introduction

There has recently been a large increase in popularity of 3D printing both in industry, education, and in the home. The continued reduction in cost of 3D printers and the simplicity of their use has contributed to 3D printed parts now being used as functional components instead of just prototype and non-stress bearing parts. This trend has also been seen in the robotics community with more and more functional components such as robot legs, fingers, wheels, and even structural frames being fabricated using standard fused deposition manufacturing (FDM) methods [1,2]. Examples of robotic systems that rely nearly completely on 3D printed ABS components include the Veter robotic vehicle [3], Shady Bot [4], the Aracna quadruped platform [5], and the Yale OpenHand Project [6].

The number and types of robotics applications within which 3D printed components can be used are limited by their low material strength, preventing roboticists from fully leveraging the promise for these rapid-prototyping techniques. In this paper, we discuss one method for greatly improving the mechanical strength of 3D printed robotic components via compositing with higher-strength resins filled into printed voids in the printed structure, retaining 3D printing's benefits of fast and easy construction and ability to make complex geometries.

Related to improving the strength of 3D printed components, researchers have developed intricate software solutions to enhance the strength of 3D printed structures through the addition of ribs and internal printed supports [7], but these finite element style methods are still limited by the

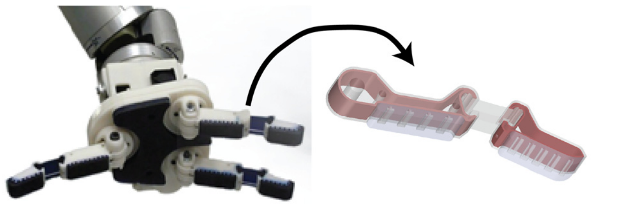

Fig. 1: Fingers of the i-HY hand [8] made using fill compositing to add strength to the 3d printed components. The red (dark) portion illustrates the internal reinforcing structure of the 3d printed part.

strength of the material being used and print orientation. 3D printing can also be useful in creating molds that are later used to cast components from stronger materials [9, 10]. This way, the advantages of 3D printing can still be utilized and result in a stronger component, made from a wider variety of materials. Casting of components can place limitations of part detail and overall geometry depending on the complexity of the mold.

Other manufacturing methods which involve both molding and machining have been used to create robotic components with additional strength. When details are required that prove too difficult to mold or cast directly, shape deposition manufacturing processes (SDM) can be used which involves sequential steps of additive molding and subtractive machining [11, 12].

Another technique used in injection molding and casting is overmolding. Here a more structural frame, typically made from metal, is placed into the mold before a polymer material is cast around it. The frame is able to enhance the strength of the part with the external details achieved through the molding process [13].

In this paper, we present a method of strengthening 3d printed parts that combines FDM 3D printing, casting, and overmolding. The original part is first 3D printed with internal voids and hollow channels. The channels and voids are then filled with an epoxy material that is stronger than the 3D printed material. The internal voids and channels act as a mold for the epoxy which hardens into an integrated reinforcing structure in the same way an overmolded metal frame would provide reinforcement from within the part. The process is similar to investment casting but the 3D printed ABS component provides the internal mold and the external detail.

In Section II, we present an overview of the strength limitation of 3D printed materials. We then present the results of flexure testing to show the increased part strength using this reinforcing technique. Finally, we show that this technique can be used to strengthen common robotic components.

This work was supported in part by the Defense Advanced Research Projects Agency (DARPA) under grand W91CRB-10-C-0141, and the Gustavus and Louise Pfeiffer Research Foundation

The authors are with the Yale University, Department of Mechanical Engineering & Material Science, New Haven, CT 06511 USA (phone: 203-432-9016; e-mail: {joseph.belter; aaron.dollar}@yale.edu).

TABLE I. RAW MATERIAL PROPERTIES OF COMMON FDM PRINTED MATERIALS AND CASTING RESINS

Material	*Tensile Strength(MPa)*	*Flexural Strength(MPa)*	*Flexural Modulus (MPa)*
ABS-P430 [17]	37.0	53.0	2,250
PLA-3052D [18]	62.0	108.0	3,600
Nylon 12 [19]	48.3	69.0	1,310
Polycarbonate [20]	68.0	104.0	2,234
Urethane 305 [15]	20.7	27.6	813
Epoxy 105/205[16]	54.5	97.2	3,178

II. STRENGTH OF 3D PRINTED PARTS

A. *Common FDM printer materials*

FDM based 3D printing relies on fusing numerous layer of material extruded from a small nozzle to form the overall part geometry. Due to this process, the available materials are currently limited to thermoplastics although additional materials with additives and blends are being investigated [14]. Table I shows the strength of the raw bulk materials most commonly used in FDM. These materials are used in the popular Stratasys® and Makerbot® brand FDM printers. As a comparison, two additional materials are shown in Table I including a common casting urethane [15] and a common two-part Epoxy resin [16]. It is important to note that these properties are the bulk properties and do not represent the properties of the material when 3D printed through FDM.

B. *Strength of material as printed*

The FDM printing method deposits fibers/beads of thermoplastic in two-dimensional layers, building up the layers on top of each other to form the desired part geometry. The layering and direction of the fibers introduces an anisotropic effect that greatly impacts overall part strength [21, 22, 23]. Numerous researcher have shown that FDM printed components show an approximate 45% decreases in modulus when compared to the bulk material [20]. Smith et al. also showed a 30-60% decrease in ultimate tensile strength based on part orientation when comparing the FDM printed test samples with the bulk material properties [20].

To verify the effects of FDM print orientation on overall part geometry, we conducted our own three-point bend testing of printed samples. The testing procedure, as shown in Fig. 2 (top left), is detailed in Section IV. All tested samples were printed from ABS-P430 [17], on a Fortus-250m printer. The print layers and fiber orientations are shown in Fig 2. (top right). In addition to the layer orientation, the print settings can be altered to print a raster infill or multiple contours per layer. Raster refers to altering the internal 45 degree hatching on the internal portion of each layer. Multiple contour settings will trace the outer perimeter inward until each layer is filled with material. Samples were also tested with sparse infill which reduces the density of the interior of the part.

It can be seen from the flexure stress curves in Fig. 2, that having the fibers oriented in the direction of stress (lengthwise down the sample) leads to greater overall strength. The samples with the layers oriented perpendicular to the direction of the stress, as in sample I from Fig.2, showed over 50% reduction in flexure stress as compared to sample IV which has all the fibers oriented parallel to the direction of stress.

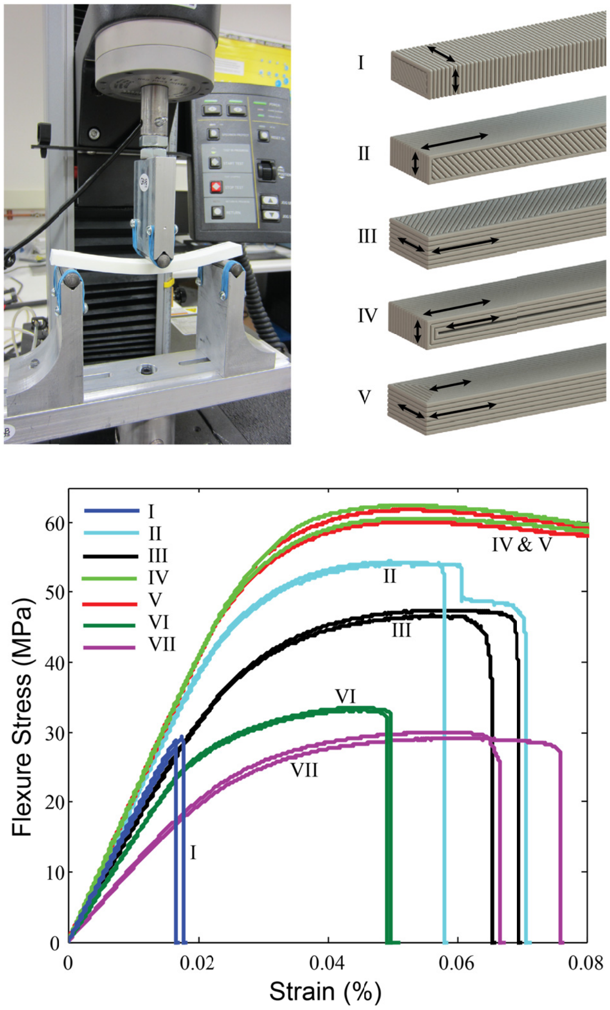

Fig. 2: The same ABS material exhibits a large variation in flexure strength based on print orientation and printer parameters. I) upright print with raster infill, II) vertical print with raster infill, III)horizontal print with raster infill, IV) vertical print with multiple contours, V) horizontal print with multiple contours, VI) sparse-fill vertical print, VII) sparse-fill horizontal print.

The effect of the print orientation and printer settings must be considered when 3D printing a component that serves structural purposes. Although for some components, the unfavorable print orientations cannot be avoided if it is required that the part to be stressed along multiple orientations. In this case, the component strength is limited by the weakest print orientation. The following section describes a method of fill compositing to improve 3D printed part strength.

III. FILL COMPOSITING TECHNIQUE

By utilizing hollow voids and channels printed internally to the components as molds for casting materials, complex internal reinforcing structures can be made that provide an increase in part strength and stiffness. Although the bulk material properties of common casting materials including urethane and epoxy, as shown in Table I, do not far exceed

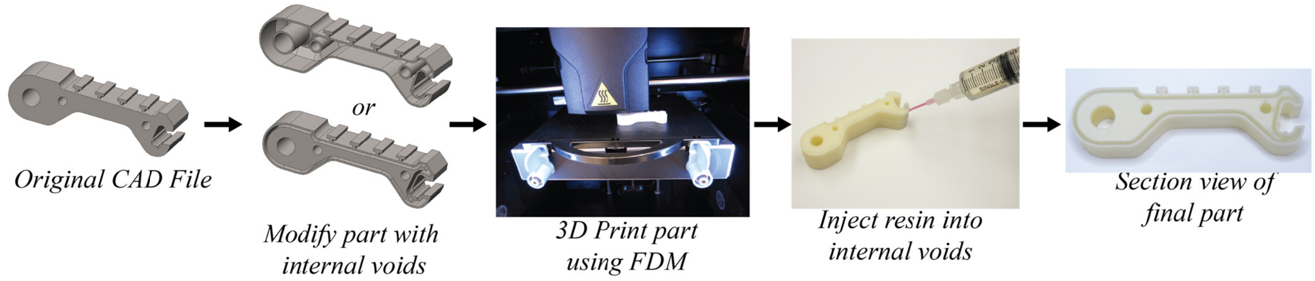

Fig. 3: The process of fill compositing uses the original part geometry but takes advantage of voids designed into the printed component which are filled with higher-strength resin. The process is illustrated here with the proximal link of the i-HY [8] robot finger.

those of the bulk 3D printed material, their properties are isotropic when molded and therefore do not exhibit the same orientation preferences as 3d printed materials. The process of reinforcing a 3D printed part with the fill compositing technique is illustrated in Fig.3.

A. Modifications to original part model

The process starts with a simple modification to the original part geometry. In Fig. 3 (far left) we show the original design of the proximal link of a robot finger. The simplest way to modify the part is to make the internal portion of the component completely hollow. A more appropriate and efficient method is to insert connected hollow regions that will best provide structural enhancement to the part based on the loading. Since the part will be 3D printed using FDM, these channels can be quite elaborate and complex. The limitations to the hollow structures are based on the ability for the printer to create these channels without the need for support material. Factors related to the specific printer including overhang angle, span, and minimum wall thickness all relate to the necessity for support stuctures. Using both a Stratasys uPrint and Stratasys Fortus-250m, the authors have successfully printed overhangs at a 30 degree angle and unsupported spans of up to 3mm without the need for support material. Also, a 0.6 mm wall thickness has shown to be sufficient to create non-porous interior and exterior layers. The FDM process makes it possible to create completely void internal structures. Other types of printing, including Z-corp powder binding and PolyJet UV curing cannot print overhangs without support material and therefore cannot produce completely hollow voids internal to the part.

B. Casting resin material into voids

The modified parts are then printed with the detailed external geometry provided by the 3D printer and internal hollow sections. A 1mm hole is drilled into the component to access the hollow cavity(s). As shown in Fig. 3, a syringe is used to inject epoxy resin into the void. The injection site should be chosen to allow for the epoxy or other casting material to set without leaking out the infill hole. Bubbles and other defects can be avoided by using a low-viscosity, slow-cure epoxy or other casting material.

C. Final part features

In the finished part, hardened epoxy provides structural reinforcement to the component from the inside. All external geometries of the original part are unchanged. The process can be compared to investment casting where the component provides the mold for the internal reinforcing cast structure.

IV. FLEXURAL TEST RESULTS

Three-point bend testing was performed to verify and quantify the increase in strength of components produced with the fill compositing technique described in Section III. The testing was performed according to ASTM-D790 [24] using an Instron® material testing system. An illustration of the testing setup is shown in Fig. 2 (top left). The loading was applied at 0.1 mm/mm/min to avoid load-rate effects.

A. Testing Samples

The flexural bend test samples were simple blocks of 8.3x 19.1 x152.4mm and were sized in accordance to the ASTM-D790 standard. All parts were printed with the same ABS-P430 material on the same Fortus-250m printer. With the same external part geometry, samples were prepared using the fill compositing technique including completely hollow printed shells filled with epoxy resin, and epoxy filled channels. Control samples were also tested including cast epoxy, and samples of identical geometry to the epoxy filled channels with printed ABS in place of the epoxy. A cross-section view of all samples is shown in Fig. 4.

B. Testing Results

The flexural stress, strain, and flexural modulus was calculated according to Eqn. 1,2,3, which assumes small angle deformation of the three point bend specimen [25].

$$\sigma = \frac{3FL}{2bd^2} \qquad \varepsilon = \frac{6dv}{L^2} \qquad E = \frac{FL^3}{4vbd^3} \qquad (1,2,3)$$

Here, σ is the flexure stress, ε is the strain, E is the flexure modulus, F is the force on the center of the beam, L is the span of the test setup (124 mm), b is the width of the sample

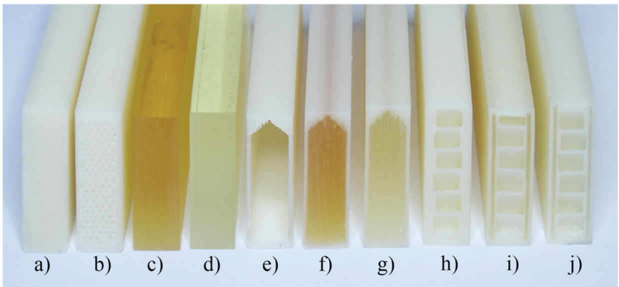

Fig. 4: Cross sections of prepared samples. a) solid printed ABS (control), b) sparse filled printed ABS, c,d) cast epoxy, e) Hollow printed shell with 30 degree overhang angle, f,g) epoxy filled ABS shell, h) printed ABS laminate structure (control), i,j) epoxy filled channels in printed ABS.

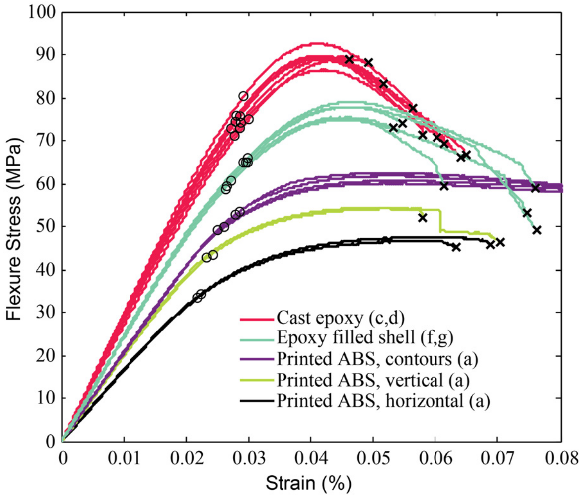

Fig. 5: Flexure strength of epoxy filled shells made using fill compositing as compared to solid printed ABS in various orientations. The test samples are labeled according to the cross section image in Fig. 4 on page 3. The black x indicates the location of failure and the black circle represents location of 0.2% yield strength.

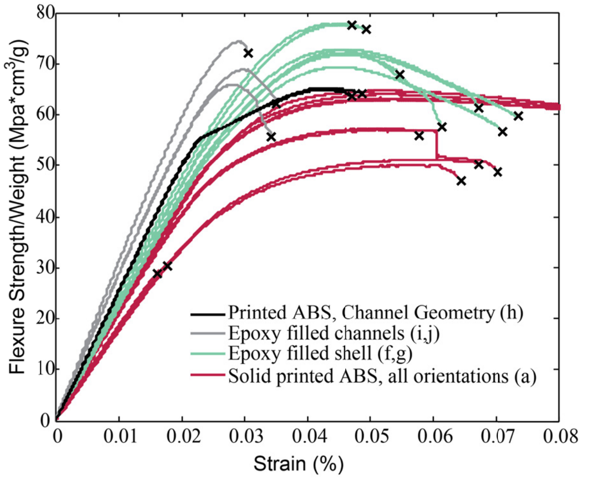

Fig. 6: Flexure strength to weight ratio of solid ABS samples to those manufactured using fill compositing.

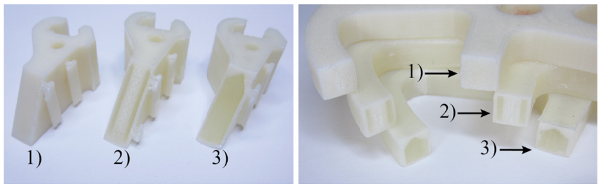

Fig. 7: Cross section view of the robotic components (left) proximal joint of the robot finger, (right) spokes and outer ring of the wheel. 1) solid printed ABS, 2) 1mm channels filled with epoxy, 3) hollow printed shell filled with epoxy.

(19.1mm) d is the sample thickness (8.3mm), v is the deflection at the center of the beam.

Fig. 5 shows a comparison of the flexure stress for samples of solid printed ABS at different orientations, as indicated by the letter label, compared to epoxy filled samples made using fill compositing. The small black circles show the point of 0.2% yield criteria, while the black x's, indicate the location of failure for the sample. Bulk samples of epoxy are also shown as a comparison. The data shows an improvement in flexure strength and flexure modulus of epoxy filled shells as compared to all orientations of solid printed ABS. Since the print orientation is known to have a large impact on the overall component strength, we also tested samples printed in the least favorable orientation (printed upright). The results show a 60% improvement in ultimate flexure strength and an improvement in overall flexure stiffness.

In addition to stronger components, many robotic applications have requirements related to reduced weight or increased stiffness. The data in Fig. 6 shows the results of three-point bend testing with the flexure stress normalized by the density of the sample. It can be seen that the epoxy filled shell samples have a higher overall strength to weight ratio than all print orientations of solid ABS. Also, the epoxy filled channel samples showed the highest possible stiffness to weight ratio of all samples tested.

TABLE II. STRENGTH AND STIFFNESS COMPARISON

Property	Solid ABS, best orientation (a)	Epoxy Filled ABS shell (f,g)	ABS printed Laminate Structure (h)	Epoxy filled channels (i,j)
Flexural Yeild Strength (MPa)	50	62	32.3	38
Flexural Modulus (MPa)	2071	2600	1475	1730
Flexural Strength/weight (Mpa*cm^3/g)	52.7	59.5	55.9	63.5
Flexural Modulus/weight ((Mpa*cm^3/g)	2150	2280	2540	2950

The results of the three-point bend testing can be summarized by comparing the solid printed ABS samples with the epoxy filled shells over all measured material properties. Table II, shows the strength and stiffness comparison of the samples. The flexural yield strength was evaluated at 0.2% offset from linear elastic behavior. The letters correspond to the sample cross-section as shown in Fig. 4.

V. ROBOTIC COMPONENT STRENGTH TESTING

Two practical examples of robotic components were manufactured to evaluate the benefits of using fill compositing as a method to strengthen 3d printed components. The first is the proximal link of a robotic finger and the second is a robot wheel whose spokes and outer perimeter were reinforced with the proposed method.

A. Testing Samples and Method

Three versions of each component were created as a comparison of actual component strength. The first was a solid printed ABS sample printed in the vertical (favorable) direction with solid raster fill. The second sample was created using fill compositing with a 1mm wide channel filled with epoxy placed just inside the entire outer perimeter of the part. For the robotic finger link, the channel was placed far enough away from the surface to maintain features used for grip pad adhesion and connection of a flexure at one end. For the wheel, the channels were placed in both the outer perimeter and spokes. The final sample was to fill the entire internal cavity of both parts with epoxy. Fig. 7 shows a cross-section view of the three sample types for

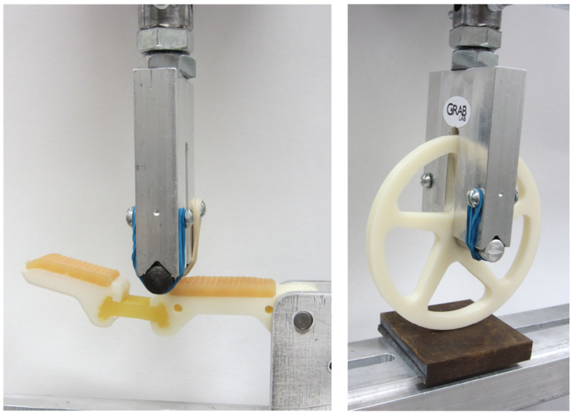

Fig.8: Images of testing setup on an Instron® Testing system to measure failure loads of the robot finger proximal joint (left) and a simple robot wheel (right).

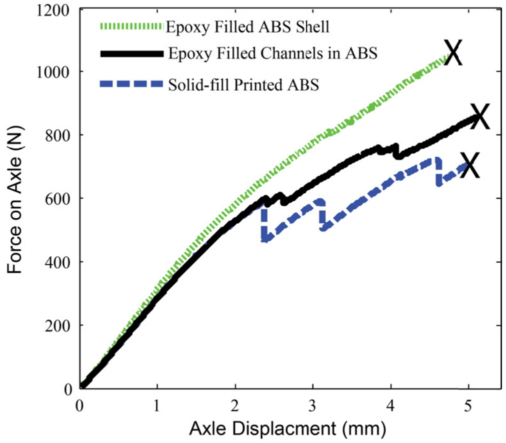

Fig. 10: Comparison of wheel strength shows a 45% increase in load capacity using fill compositing with epoxy resin. The black X shows the point of failure.

the proximal finger link and the wheel. You will notice in the hollow printed shell filled with epoxy, the upper surface was tapered to provide a 30 degree overhang angle since the span was too wide to bridge otherwise with the FDM printer.

The overall strength of the samples was tested using a modified three point bend fixture. For the proximal finger link, a load was placed on the distal end of the proximal linkage and applied until failure. The wheel strength was tested by applying a load to the center axle against a flat plate. All three sample types were oriented in the same spoke angle during the test as illustrated in Fig. 8 (right).

B. Testing Results

Due to the complex geometry being tested, we will directly compare failure load instead of failure stress as we did in the standard three-point bend tests. Fig. 9 shows the comparison of proximal robot link strength (to bending in the extension direction) of the three samples types. Fig. 10 shows the comparison of robot wheel strength between solid printed samples and those filled with resin. These plots also show the relative stiffness of the three samples by analyzing the slope of the force, displacement curve. The results of the component tests can be best summarized in Table III.

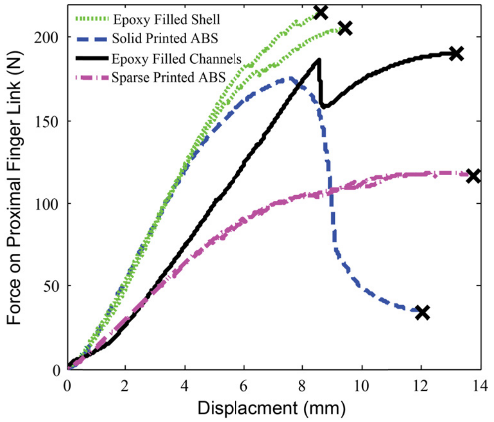

Fig. 9: Comparison of robotic finger link strength shows improvement in failure strength using a 3d printed shell of the same part geometry filled with epoxy resin. The black X shows the point of failure.

VI. Discussion

We have shown in this paper that we can enhance the strength of 3d printed components above the capabilities of the solid printed material even in the most preferable print orientation. The process of fill compositing is simple and takes advantage of the benefits of low-cost FDM printing. This has a direct effect on the future use of 3D printed parts in the robotics community.

In the three-point bend samples, the overall yield strength of a simple printed hollow structure filled with epoxy resin was 24% higher than the most preferable solid ABS print orientation. The stiffness was also 25% higher with the Epoxy filled samples. One of the greatest advantages was the improvement in strength and stiffness to weight ratio of 13.6% and 16.1% respectively, through the use of hollow channels designed into the part and filled with epoxy resin.

The robotic components also showed improved properties through the use of fill compositing. The finger link showed a 19% improvement in failure load, while the wheel showed a 45% increase in failure load. The investigation into the preferred print orientation showed that the strength limitations of the worst print orientations can be overcome using fill compositing.

TABLE III. Strength Comparison of Robotic Components

Property	Solid ABS orientation (a)	Epoxy Filled ABS shell (c)	Epoxy filled channels (b)
**Finger Link**			
Peak Force (N)	175	208	190
Stiffness (N/mm)	30.6	32.7	26.1
**Wheel**			
Peak Force (N)	720	1048	895
Stiffness (N/mm)	268.7	303.7	269.5

There still was a significant improvement in the strength of even the epoxy filled printed shells when the shells were printed in the preferred orientation. This shows that it is still beneficial to consider the orientation of the print fibers when using this technique to strengthen 3D printed parts.

One limitation to the fill compositing method is the necessity for the parts to be printed with non-porous internal voids. Some FDM printer settings will create porous parts that do not properly block flow of the resin into sparse fill areas of the part. It is necessary to adjust printer setting, specifically with regard to raster fill, to prevent porous cavity surfaces.

Within this investigation of fill compositing, the authors have focused on the use of Epoxy resin as the compositing material of choice. In future work, the authors plan to investigate more materials including extremely tough urethanes, and resin additives including chopped glass fibers. The bond strength between the filled resins and the ABS printed material will also be studied.

Acknowledgment

The authors would like to thank undergraduate research assistants Peter Nguyen, Hannah Mae Robinson, Alex Tenn, and Sagar Yadama for their effort on conducting material testing and creating samples used in this work.

References

[1] Won, Jey, Kathryn DeLaurentis, and Constantinos Mavroidis. "Rapid prototyping of robotic systems." Robotics and Automation, 2000. Proceedings. ICRA'00. IEEE International Conference on. Vol. 4. IEEE, 2000.

[2] De Laurentis, Kathryn J., Fung Felix Kong, and Constantinos Mavroidis. "Procedure for rapid fabrication of non-assembly mechanisms with embedded components." Proceedings of DETC. 2002.

[3] Veter- robotics vehicle for researcher and makers, accessed Feb, 2014 at veterobot.com

[4] Marsette Vona, Carrick Detweiler, Daniela Rus. Shady: Robust Truss Climbing With Mechanical Compliances, International Symposium on Experimental Robotics, pp. 431–440, July, 2006

[5] Sara Lohmann, Jason Yosinski, Eric Gold, Jeff Clune, Jeremy Blum, and Hod Lipson. Aracna: An open-source quadruped platform for evolutionary robotics. In Proceedings of the 13th International Conference on the Synthesis and Simulation of Living Systems, 2012.

[6] Raymond R. Ma, Lael U. Odhner, and Aaron M. Dollar, "A Modular, Open-Source 3D Printed Underactuated Hand", proceedings of the 2013 IEEE International Conference on Robotics and Automation (ICRA 2013), Karlsruhe, Germany, May 6-10, 2013.

[7] Stava, Ondrej, et al. "Stress relief: improving structural strength of 3D printable objects." ACM Transactions on Graphics (TOG) 31.4 (2012): 48.

[8] Lael U. Odhner, Leif P. Jentoft, Mark R. Claffee, Nicholas Corson, Yaroslav Tenzer, Raymond R. Ma, Martin Buehler, Robert Kohout, Robert D. Howe, and Aaron M. Dollar, "A Compliant, Underactuated Hand for Robust Manipulation", International Journal of Robotics Research, 2014, (in press)

[9] Jamalabad, V. R., Whalen, P. J., Pollinger, J., Agarwala, M. K., & Danforth, S. C. (1996, August). Gelcast molding with rapid prototyped fugitive molds. In Proceedings of Solid Freeform Fabrication Symposium, Austin, TX.

[10] Zonder, L., Sella, N., "Precision Prototyping, The role of 3D printed molds in the injection molding industry", White Paper by Stratasys Inc, 2013, available at http://www.stratasys.com/~/media/Main/Files /White%20Papers/SSYS-WP-InjectionMolding-9-23-13.pdf

[11] Cham, J. G., Bailey, S. A., Clark, J. E., Full, R. J., & Cutkosky, M. R. (2002). Fast and robust: Hexapedal robots via shape deposition manufacturing. The International Journal of Robotics Research, 21(10-11), 869-882.

[12] Cooper, A. G., Kang, S., Kietzman, J. W., Prinz, F. B., Lombardi, J. L., & Weiss, L. E. (1999). Automated fabrication of complex molded parts using mold shape deposition manufacturing. Materials & design, 20(2), 83-89

[13] Dickie et al. "Apparatus for Plastic Injection Overmolding" US Patent # 5,182,032, Jan. 1993

[14] Duty, Chad. "Carbon Fiber Reinforced Polymer Additive Manufacturing." Oak Ridge Carbon Fiber Composites Consortium (2012).

[15] Smooth-on Technical Documentation, "Smooth-Cast® 300 Series, Bright White, Ultra Low Viscosity Liquid Plastics", Accessed Feb 2014, Available at http://www.smooth-on.com/tb/files/Smooth-Cast_300q,_300,_305___310.pdf

[16] West System Technical Documentation, "Technical Data Sheet, 105 Epoxy Resin / 205 Fast Hardener", Gougeon Brothers Inc. Available at http://www.westsystem.com/ss/assets/Product-Data-PDFs/TDS%20105_205.pdf

[17] Stratasys Technical Documentation "ABSplus-P430, Production-Grade Thermoplastic for Dimension 3d Printers", 2013, available at http://www.stratasys.com/~/media/Main/Secure/Material%20Specs%20MS/Fortus-Material-Specs/Fortus-MS-ABSplus-01-13-web.ashx

[18] Ingeo Technical Bulletin, NatureWorks LLC, "Ingeo resin product guide", 2011, available at http://www.natureworksllc.com/~/media/Technical_Resources/one-pagers/ingeo-resin-grades-brochure_pdf.pdf

[19] Stratasys Technical Documentation, "FDM Nylon 12, Production-Grade Thermoplastic for Fortus 3d Production Systems", 2013, available at http://www.stratasys.com/~/media/Main/Secure /Material%20Specs%20MS/Fortus-Material-Specs/Fortus%20 Nylon12%20Material%20SS%20EN%2012-13%20Web.pdf

[20] Walter Castro Smith, and Richard W. Dean, "Stuctural characteristics of fused deposition modeling polycarbonate material", Polymer Testing, 2013, Elsevier Ltd.

[21] Lee, C. S., Kim, S. G., Kim, H. J., & Ahn, S. H. (2007). "Measurement of anisotropic compressive strength of rapid prototyping parts." Journal of Materials Processing Technology, 187, 627-630.

[22] El-Gizawy, A. Sherif, Shan Corl, and Brian Graybill. "Process-induced Properties of FDM Products." Proceedings of the ICMET, International Conference on Mechanical Engineerings and Technology Congress & Exposition (2011).

[23] Laliberte, Thierry, Clement M. Gosselin, and Gabriel Cote. "Practical prototyping." Robotics & Automation Magazine, IEEE 8.3 (2001): 43-52.

[24] ASTM D790, "Standard test method of flexural properties of unreinforced and reinforced Plastics and electrically insolating materials", ASTM.org, ASTM International

[25] Hibbler, R.C., *Statics and Mechanics of Materials, second edition*. NJ: Pearson-Hall Inc. 2004
